# Use of cardiac cell cultures from salmonids to measure the cardiotoxic effect of environmental pollutants

**DOI:** 10.1111/jfd.14018

**Published:** 2024-09-29

**Authors:** Torben Krebs, Julia Bauer, Sarah Graff, Lukas Teich, Markus Sterneberg, Marina Gebert, Henrike Seibel, Bettina Seeger, Dieter Steinhagen, Verena Jung‐Schroers, Mikolaj Adamek

**Affiliations:** ^1^ Fish Disease Research Unit, Centre for Infection Medicine University of Veterinary Medicine Hannover Hannover Germany; ^2^ Working Group Fish Health and Welfare, Section Aquaculture and Aquatic Resources Fraunhofer Research Institution for Individualized and Cell‐Based Medical Engineering IMTE Lübeck Germany; ^3^ Institute for Food Quality and Food Safety University of Veterinary Medicine Hannover Hannover Germany

**Keywords:** cell cultures, crude oil, heart, microplastic, nanoplastic

## Abstract

Environmental stressors such as micro‐ and nanosized plastic particles (MNPs) or crude oil have a detrimental effect on aquatic animals; however, the impact upon the cardiovascular system of fish remains relatively under‐researched. This study presents a novel approach for investigating the effect of crude oil and MNPs on the cardiac system of fish. We used salmonid larvae and cardiac cell cultures derived from hearts of salmonid fish and exposed them to environmental stressors. Following exposure to plastic particles or crude oil, the larvae exhibited some variation in contraction rate. In contrast, significant alterations in the contraction rate were observed in all cardiac cell cultures. The greatest differences between the control and treatment groups were observed in cardiac cell cultures derived from older brown trout. Following 7 days of exposure to MNPs or crude oil in Atlantic salmon larval hearts or cardiac cell cultures, there were only minor responses noted in mRNA expression of the selected marker genes. These findings show the use of a novel in vitro technique contributing to the existing body of knowledge on the impact of MNPs and crude oil on the cardiovascular system of salmonids and the associated risk.

## 1 INTRODUCTION

Anthropogenic activities are having a negative impact on aquatic ecosystems worldwide, leading to a decline in biodiversity (Aronson et al., 2011). One of the drivers of this negative impact is the use of fossil resources and the products derived from them. Some scientists refer to the last few decades as the Plasticocene, in which environmental pollution is caused by various petroleum‐derived components, including plastic particles, synthetic materials and crude oil, which can have a particularly negative impact on aquatic life (Chen et al., 2024; Sørhus et al., 2021).

Crude oil is considered to be one of the most significant pollutants entering the water, and it has a massive impact on marine ecosystems. Several oil spills have occurred worldwide in recent decades (Chen et al., 2019; Martínez‐Gómez et al., 2006). In 2010, the largest oil spill in history occurred in the Gulf of Mexico, the so‐called Deepwater Horizon oil spill, in which around 4.4 million barrels of oil leaked into the sea (Murawski et al., 2020). Exposure to oil has been found to impair cardiac function and development, leading to disruption of heartbeat and influencing heart cell physiology in embryos of almost all fish species studied in detail (Incardona & Scholz, 2016). Researchers discovered that offshore fish populations were in a lymphogenic or immunocompromised state after being exposed to polycyclic aromatic hydrocarbons (PAHs) following the disaster (Ali et al., 2014). Crude oil is composed of thousands of different components, some of which may be responsible for the observed toxicity (Meador & Nahrgang, 2019). Several other studies have also demonstrated the effects of oil spills on increased fish mortality (Fodrie et al., 2014; Hjermann et al., 2007).

Other common pollutants are plastic particles. Plastics are polymeric materials produced by polymerization of monomers derived from fossil resources, such as oil or coal (Kim et al., 2020). Global production of plastics reached around 400.3 million tonnes in 2022, representing an extremely large increase over the last 70 years (PlasticsEurope, 2023). Many different sources transport plastic into the aquatic environment (Thompson, 2017), where it remains for months up to several thousands of years due to its low biodegradability (Barnes et al., 2009). It is estimated that around 20 million tonnes of plastic enter the oceans each year (Borrelle et al., 2020) and there is no indication that the production of synthetic polymers made from crude oil will decrease in the future (Akdogan & Guven, 2019; Mendoza & Balcer, 2019). Through intense oxidation under the exposure of solar UV radiation macroplastics degrade into secondary microplastics (MPs) and nanoplastics (NPs) (Andrady, 2022). MPs are plastic particles that are smaller than 5 mm (Browne et al., 2007). The definition of NPs varies among scientists. Some define NPs as smaller than 1000 nm (Gigault et al., 2018), while others consider only those smaller than 100 nm in size (Besseling et al., 2019).

MPs and NPs are generally known to have toxic effects on fish and other aquatic organisms (Torres‐Ruiz et al., 2021). NPs inhibit the growth of microalgae (Yang et al., 2021) and induce oxidative stress and metabolic disorders in scallops (Sun et al., 2024). In addition, studies have investigated the toxic effects of MPs and NPs on human and animal cell lines (Fu et al., 2024; Huang et al., 2024; Wu et al., 2024). For example, research has shown that human embryonic stem cells can absorb polystyrene NPs, which can compromise the efficiency of cardiomyocyte differentiation (Li et al., 2024). Fish toxicology studies were primarily conducted on zebrafish, as they can be used to combine genetic, cellular and whole‐organism endpoints (Pitt, Kozal, et al., 2018; Torres‐Ruiz et al., 2021). However, the research and the results on the effects and hazards of crude oil and micro‐ and nanoplastics (MNPs) on the cardiovascular system of fish are inconsistent and not sufficiently detailed due to a lack of suitable techniques and meaningful measurement methods. Studies have shown that when polystyrene particles are injected into the yolk of zebrafish embryos, the particles accumulate around the heart, suggesting that they could affect this organ (Pitt, Kozal, et al., 2018; Pitt, Trevisan, et al., 2018; Veneman et al., 2017). Several studies have investigated the impact of NPs on fish heart rate, with results ranging from no change to bradycardia (Santos et al., 2024; Sun et al., 2021; Zhou et al., 2023).

In the present study, we introduce novel tools and techniques for investigating the effect of crude oil and MNPs on the cardiac system of fish. This was done by monitoring the heart contraction rate of salmonid larvae from Atlantic salmon *Salmo salar* (Linnaeus 1758), brown trout *Salmo trutta fario* (Linnaeus 1758) and rainbow trout *Oncorhynchus mykiss* (Walbaum 1792). In addition, we have developed and implemented a new in vitro technique in which contracting cardiac cell cultures of these three species are used to study the toxic effects of the aforementioned substances (Graff et al., in preparation; based on a protocol from (Sander et al., 2013)). The non‐feeding, 1‐week post‐hatching yolk‐sac larvae and cell cultures derived from them were exposed to varying concentrations of crude oil and MNPs for a 7‐day period, with their contraction rates subsequently quantified and compared. Additionally, the same experiment was conducted using cardiac cell cultures from 11‐month‐old brown trout to compare the results with those obtained from larvae. Moreover, immunocytochemistry was conducted to confirm the isolation and cultivation of cardiomyocytes. Furthermore, gene expression analysis was performed on selected gene markers for antioxidant enzymes, pro‐inflammatory processes and crucial mediators within the immune system, which had been previously observed to be regulated in response to exposure to environmental stressors on day 7 post‐exposure in Atlantic salmon larvae and cardiac cell cultures using RT‐qPCR.

## 2 MATERIALS AND METHODS

### 2.1 Fish and larvae husbandry

For the experiments, we utilized non‐feeding, one‐week post‐hatching salmonid larvae in the yolk‐sac stage and 11‐month old brown trout of various origins. Atlantic salmon eggs were provided by the State Agency for Nature, Environment and Consumer Protection (LANUV) located in the German state of North Rhine‐Westphalia. Brown trout and rainbow trout eggs, as well as adult brown trout were obtained from several commercial fish farms in Germany. Eggs were obtained in the eyed stage for all species and were hatched at the Fish Disease Research Unit, University of Veterinary Medicine Hannover (Figure S1). The larvae as well as the older brown trout were maintained in a flow through aquaculture system at 10–12°C and with suitable aeration. The research was conducted on non‐feeding larvae and tissues collected from fish post‐mortem in accordance with local and international animal experimentation legislation (Article 4, TSchG Killing for scientific purposes, Annunciation number: TiHo‐T‐2022‐3).

### 2.2 Cell cultures

Beating cell cultures were prepared from salmonid hearts by modifying the protocol for primary cardiomyocytes from zebrafish hearts (Sander et al., 2013). Two hundred and ten hearts from healthy looking non‐feeding, yolk sac larvae were used for each 24‐well plate, with three replicates of 70 hearts each. For cell cultures from 11‐month‐old brown trout 18 hearts were used for each 24‐well plate, with three replicates of 6 hearts each. To extract the heart, larvae and fish were anaesthetized using MS‐222 (PHARMAQ). Extracted hearts (Figure S2) were placed in a petri dish filled with heparin buffer, containing 1x PBS (Sigma), 10 U/mL heparin (Sigma), and 100 U/mL penicillin/streptomycin (Sigma), and then placed on ice. The hearts were washed several times by pipetting up and down using a 10 μL pipette or a 1 mL syringe to remove erythrocytes. After washing with the heparin buffer, the hearts were transferred to a 2 mL Eppendorf tube containing 1 mL perfusion buffer made up of 1× PBS (Sigma), 10 mM HEPES (Sigma), 30 mM taurine (Sigma), 5.5 mM glucose (Sigma), and 10 mM 2,3‐Butanedione monoxime (Sigma). Hearts were washed two to three times in the perfusion buffer by centrifugation (300 × g, 4°C, for 5 min) to eliminate residual erythrocytes (Figure S3). Subsequently, the perfusion buffer was eliminated and replaced with digestion buffer comprising 950 μL perfusion buffer, 50 μL CaCl_2_ 1 mM (Sigma), 5 mg collagenase type II (Worthington Biochemical), and 5 mg collagenase type IV (Worthington Biochemical) for each 1 mL. Digestion was conducted at 25°C, 900 rpm for 90 min with a thermomixer. Occasionally, the digestion buffer was mixed with the hearts by pipetting the buffer up and down. During the digestion process, 24‐well plates were coated with fibrin gel prepared from fibrinogen (Sigma), thrombin (Sigma) and Advanced DMEM/F12 (ThermoFisher Scientific) plating medium. The complete digestion of the hearts was checked both visually and through the use of a pipette. The digestion was deemed successful when no further cellular clumps were observed and the 10 μL pipette tip exhibited no blockage. After complete digestion of the hearts, the cardiomyocytes were washed with stopping buffers 1 to 7 as described in Sander et al. (2013). After the final wash, the cell pellet was resuspended in advanced DMEM/F‐12 plating medium (ThermoFisher Scientific) supplemented with 2 mM GlutaMAX (ThermoFisher Scientific), 20% fetal calf serum (Sigma), and 100 U/mL Penicillin/Streptomycin (Sigma) and 1 mL/500 mL Normocin (InvivoGen) per 500 mL of medium was utilized. Furthermore, for new cultures, the plating medium was supplemented with recombinant human Epidermal Growth Factor (0.5 ng/mL) (PeproTech), recombinant human Basic Fibroblast Growth Factor (2 ng/mL) (PeproTech) and recombinant human Insulin (5 μg/mL) (Sigma). During the first week, contraction‐inhibitor BDM was added to the plating medium to ensure cell attachment to the fibrin gel. Cardiomyocytes were then plated in each well containing 500 μL of medium and incubated at 15°C and 2% CO_2_. Weekly, 300 μL of medium was removed and replaced with an equal volume of freshly prepared medium. Following a period of 3–4 weeks during which the cell cultures were grown, evidence of cell contraction in each well was observed, indicating that the cultures were ready to be employed in the planned experiments (Figure S4).

### 2.3 Exposure of salmonid larvae to environmental stressors

All three species of salmonid larvae were exposed to MNPs in different concentrations for an observation period of 7 days. Additionally, a different set of larvae was exposed to crude oil in different concentration. Twenty healthy looking non‐feeding yolk‐sac larvae each were kept in 250 mL cell culture flasks at 12°C water temperature with suitable aeration. For the plastic experiments, three different concentrations of either 0.05 μm nanoplastic particles (Polysciences) or 6 μm microplastic particles (Polysciences) were used (0.1, 1 and 10 ppm). The concentrations of plastic particles were based on the findings of previous studies on the effects of such particles on the heartbeat of zebrafish larvae (Pitt, Kozal, et al., 2018). Additionally, per experiment one control group without plastic particles was added. For the crude oil experiments, two different concentrations of crude oil (Sigma) in freshwater were used (0.1 and 1 g/L) based on previous studies on larval or juvenile fish (Couillard et al., 2005; Ramachandran et al., 2004; Schein et al., 2009). Prior to the experimental procedure, the water‐accommodated fraction (WAF) of the crude oil was freshly prepared by stirring for a period of 24 h in room temperature to ensure thorough mixture. Subsequently, the oil‐in‐water solution was added to the vessels with larvae or cell cultures. Here, a control group without the addition of crude oil was added as well. Sampling for all experiments was done immediately after exposure and on day 1, 3, 5 and 7 post exposure. Per each day of sampling the heart rate/contraction rate, defined by the number of heartbeats for 30 s, was recorded for four larvae per group and evaluated under the fluorescence microscope. Following the recording of the heartbeat, the larvae were euthanized by an overdose of MS‐222 (0.5 g/L) and then stored in RNA‐Later (Sigma) at minus 80°C. Prior to the gene expression analysis, the larval hearts were dissected and placed in TRI‐Reagent (Sigma).

### 2.4 Exposure of cardiac cell cultures from salmonids to environmental stressors

Three‐ to four‐week‐old contracting cell cultures from larvae from all three salmonid species as well as contracting cell cultures from older brown trout were exposed to nano‐ and microplastics and to crude oil in the same concentrations as above in a similar experimental set‐up. Stock solutions containing plastics or crude oil were added to the prepared advanced DMEM/F‐12 plating medium to achieve the appropriate concentrations. Before adding the environmental stressors into the cells, each well was examined to ascertain the uniformity of the contraction. Cell cultures were incubated at 15°C and 2% CO_2_. Sampling was performed immediately after exposure and on day 1, 3, 5 and 7 post exposure. Contractions of the heart cell cultures were also recorded for 30 s and evaluated under a fluorescence microscope to ensure comparability. Evaluation of the contractions was performed by using an ImageJ tool called *Myocyter*. In addition, three samples per time point and treatment group from Atlantic salmon larvae cardiac cell cultures were collected in TRI reagent (Sigma) for gene expression analysis.

### 2.5 Molecular biology analysis

RNA was isolated from the heart cell culture samples as well as from heart tissue from exposed larvae on day 7 post exposure and total RNA was extracted using TRI‐Reagent (Sigma) in accordance with the manufacturer's instructions. cDNA was transcribed from upto 250 ng total RNA using the Maxima First Strand cDNA Synthesis Kit (Thermo Fisher Scientific). cDNA samples were diluted 1:20 with nuclease‐free water prior to quantitative RT‐PCR analysis.

RT‐PCR was performed in duplicate using Maxima SYBR Green 2× Mastermix (Thermo Fisher Scientific) on a StepOne Thermocycler (Applied Biosystems). The reaction mix contained 1× Maxima SYBR Green Mastermix (containing 10 nM ROX), 0.2 μM of each primer and 3.0 μL cDNA (1:20 dilution). Nuclease‐free water was added to a final volume of 10 μL. The amplification programme consisted of an initial denaturation phase of 10 min at 95°C, 40 identical cycles of denaturation for 30 s at 95°C, annealing at 55°C for 30 s and a final elongation at 72°C for 30 s. Measurements were normalized by determination of three reference genes (coiled‐coil domain‐containing protein 84, elongation factor 1α and 39S ribosomal protein L40). For quantification, copy numbers of gene‐specific RNA were normalized against 10^5^ copies of the reference gene rainbow trout elongation factor 1α (*ef1α*/100,000), 10^3^ copies of the reference gene Atlantic salmon coiled‐coil domain‐containing protein 84 (*coilp84*/1000) and 10^3^ copies of the reference gene Atlantic salmon 39S ribosomal protein L40 (*39SL40*/1000). The targeted genes were selected based on the existing literature. In the studies, the genes were observed to be regulated in response to exposure to environmental stressors. Catalase (CAT), Glutathione peroxidase 1 (Gpx1) and superoxide dismutase 2 (SOD2) are one of the main antioxidant enzymes. The cytokines interleukin 1b (IL‐1b), interleukin 6 (IL‐6 L) and interleukin 8 (IL‐8) are all involved in the pro‐inflammatory process. Suppressors of cytokine signalling 1 (SOCS1) and 3 (SOCS3) are crucial mediators in both the innate and adaptive immune systems, as well as regulators of the JAK/STAT signalling pathway. Primer sequences of the selected genes are shown in Table S1.

### 2.6 Immunocytochemistry

For fluorescence immunocytochemistry (FICC), the additional cell cultures were grown on collagen‐coated glass slide inserts and fixed with 4% buffered PFA for 10 min. After washing twice with PBS, the cultures were blocked with 1% bovine albumin fraction V (Roth, Germany) and 0.5% saponin (Sigma, Germany) for 30 min. After two washes with PBS, the cultures were stained overnight at 4°C with 1:300 diluted anti‐alpha‐sarcomeric actin antibody (Monoclonal Anti‐Actin α‐Sarcomeric antibody clone: 5C5, Sigma) or anti‐tropomyosin antibody (Monoclonal Anti‐Tropomyosin antibody clone: TM311, Sigma). After two washes, the goat anti‐mouse Alexa488 conjugated secondary antibody (ThermoFisher Scientific) (diluted 1:1000 in PBS) was added and the cultures were incubated in the dark for 1 h. After two washes, the cultures were mounted with Roti‐Mount PI and observed under a fluorescence microscope (Zeiss, Germany) and digitized using a Samsung SM‐G965 camera. Two types of isotype controls were included: (i) the primary antibody was replaced by the blocking solution, and (ii) the primary antibody was replaced by control mouse IgG (Thermo Fisher Scientific).

### 2.7 Statistics

Statistical analyses were performed using Systat software SigmaPlot 12. Log10 data of the contractions were tested for normal distribution and equality of variances. Significant differences (*p* < .05) between control and different treatments (*) were assessed using a two‐way analysis of variance (ANOVA), followed by pairwise multiple comparisons using the Holm‐Sidak method if the data showed a normal distribution. Gene expression data (Log10) were tested with a one‐way ANOVA. If the data were not normally distributed, a Kruskal‐Wallis one‐way ANOVA on ranks was used. Significant differences (*p* < .05) between control and different treatments are pointed out by a *.

## 3 RESULTS

### 3.1 Effect of MNPs and crude oil on cardiac contractions of salmonid larvae

**FIGURE 1:**
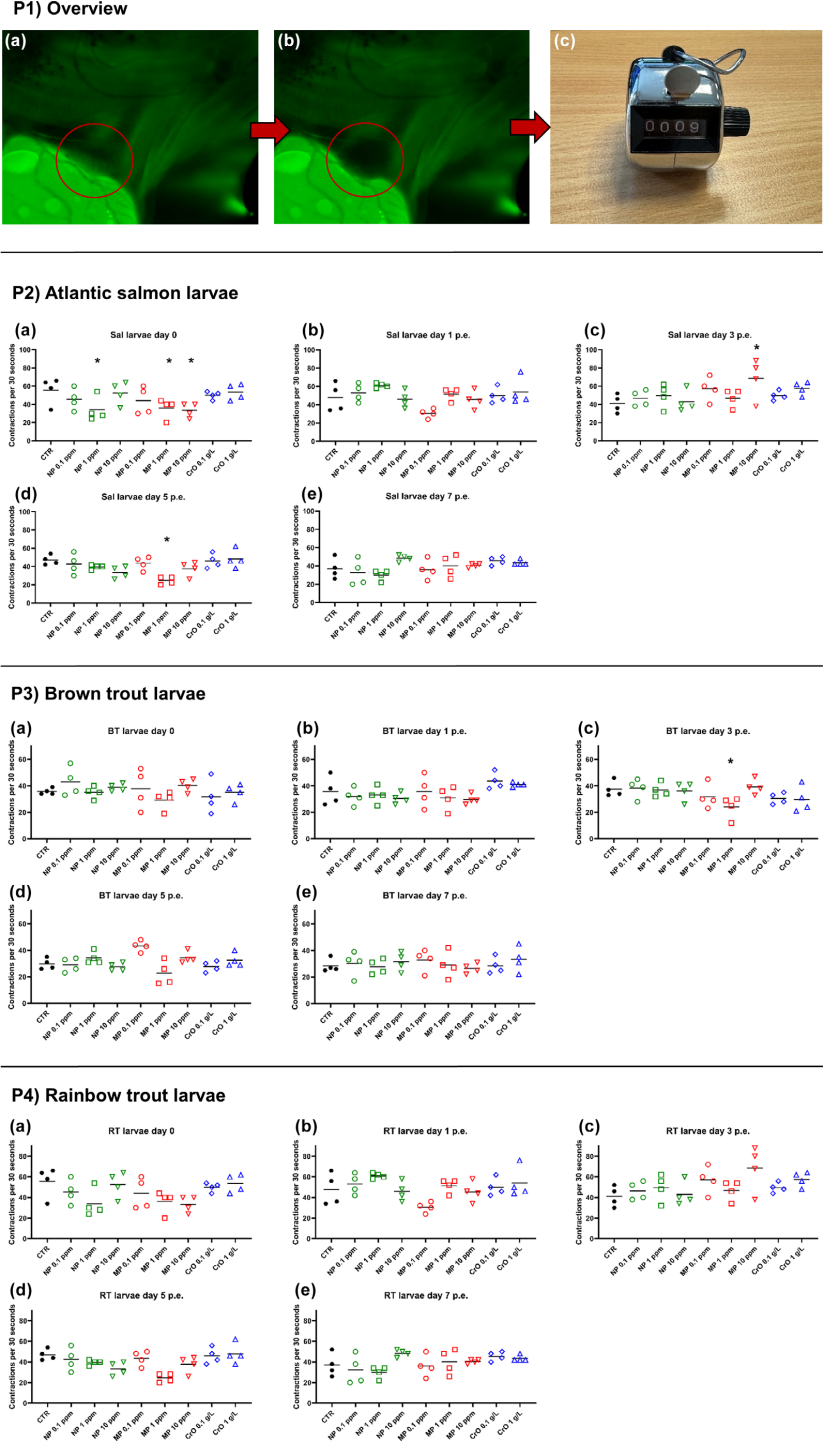
Heart rate in Atlantic salmon (Panel P2), brown trout (Panel P3) and rainbow trout (Panel P4) larvae during exposure to microplastic particles (MP), nanoplastic particles (NP) and crude oil (CrO) at different concentrations. Non‐exposed larvae are treated as control (CTR). The overview (Panel P1) provides a definition of a heartbeat and an illustration of the method of counting it. A heartbeat is defined as the transition from a state of the heart in which the blood is absent (P1a) to a state in which the blood is present (P1b), and then back to a state of the heart in which the blood is absent. The red circles indicate the position and the state of the heart, which may be either bloodless (clear appearance of the heart) (P1a) or blood‐filled (dark appearance of the heart) (P1b). The number of heartbeats was counted manually with a hand counter (P1c). Results are presented as scatter plots of the number of heartbeats per 30 s with all data points and the mean shown. * Indicates statistically significant differences in cardiac contractions of exposed larvae compared to unexposed controls within the time point at *p* < .05. Statistical analysis was performed using two‐way ANOVA, with multiple comparison test performed using the Holm‐Sidak method.

The hand counted heart rate varied in non‐exposed larvae between 22 and 68 contractions per 30 s. The exposure of the larvae to plastic particles or crude oil induced some variation in the contraction rate, but there were only a few significant changes in the heart rate of the salmonid larvae during the observation period. The most significant differences between the control and treatment groups were observed in Atlantic salmon larvae (Figure 1, Panel P2a–e). Differences were found in the treatment groups 1 ppm NP (*p* = .02), 1 ppm MP (*p* = .047), and 10 ppm MP (*p* = .023) directly after exposure to the environmental stressors (Figure 1, Panel P2a). In all three cases, the heart rate was lower compared to the control group, but it returned to the contraction rate of the control group by day 1 p.e. On day 3 post‐exposure, the heart rate was significantly higher (*p* = .029) in Atlantic salmon larvae treated with 10 ppm MP compared to the control (Figure 1, Panel P2c). On day 5 post‐exposure, the heart rate of this treatment group returned to the level of the control group, but then the heart rate of the 1 ppm MP treatment group was significantly lower compared to the control (*p* < .001) (Figure 1, Panel P2d). In brown trout larvae (Figure 1, Panel P3a–e), the heart rate remained largely unchanged after exposure to plastic particles or crude oil, with a significant change observed only on day 3 post‐exposure. Then, the heart rate of brown trout larvae treated with 1 ppm MP was significantly lower compared to the control (*p* = .025) (Figure 1, Panel P3c). In rainbow trout larvae, exposure to plastic particles or crude oil induced some variation, but no significant changes in heart rate (Figure 1, Panel P4a–e). No mortality or accumulation of MNPs inside the body was observed during the experimental trial.

### 3.2 Effect of MNPs and crude oil on contractions of cardiac cell cultures from salmonid larvae and older brown trout

**FIGURE 2:**
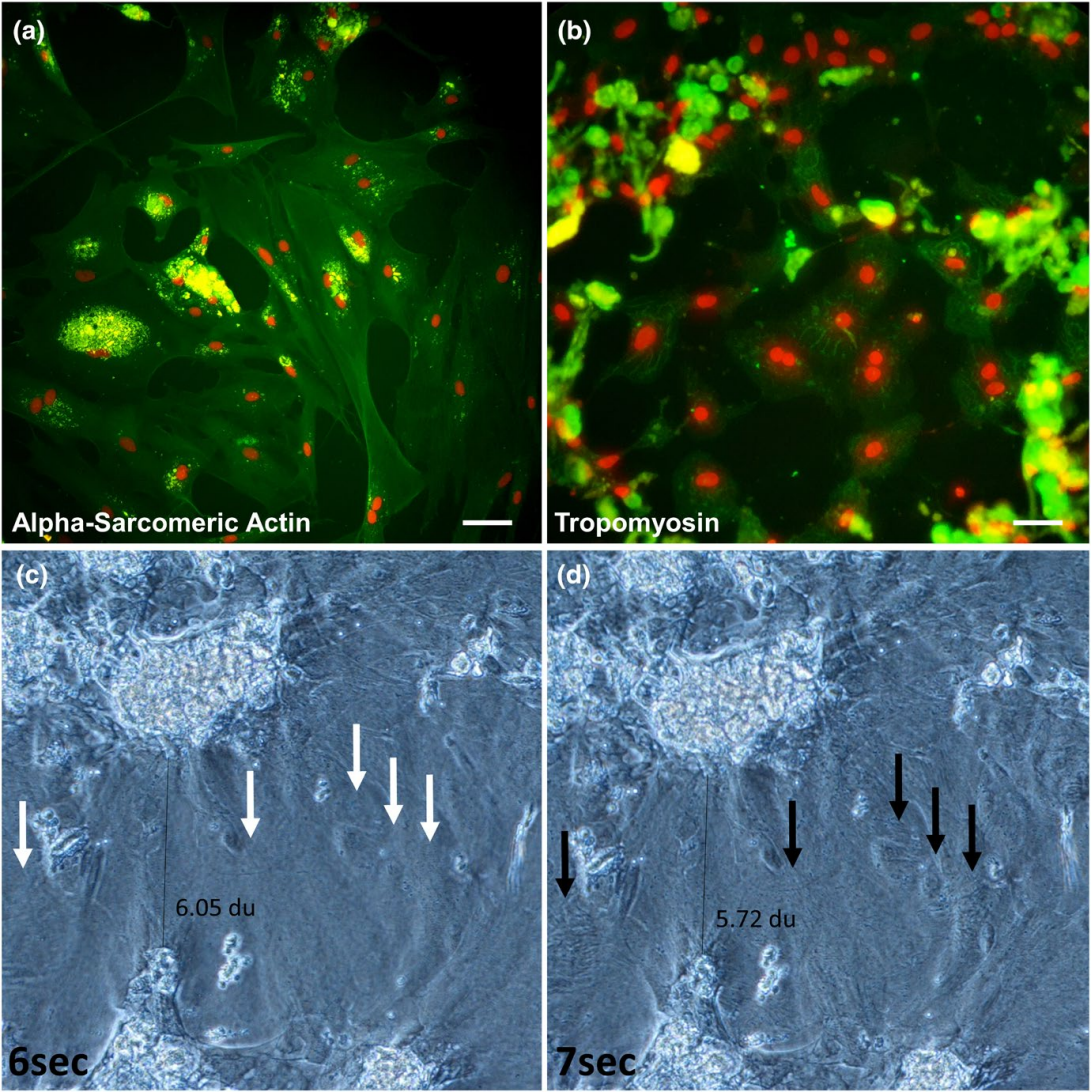
Cardiac cell cultures from rainbow trout. (a) Immunocytochemistry for alpha‐sarcomeric actin (green), nuclei (red) residual autofluorescence (yellow). (b) Immunocytochemistry for tropomyosin (green), nuclei (red) residual autofluorescence (yellow). (c and d) frames from Video S1 showing the non‐contracted (c) and contracted culture (d), note the areas of contraction marked with white and black arrows and the reduction in distance (indicated by distance units—du) between the dead cell aggregates during contraction.

**FIGURE 3:**
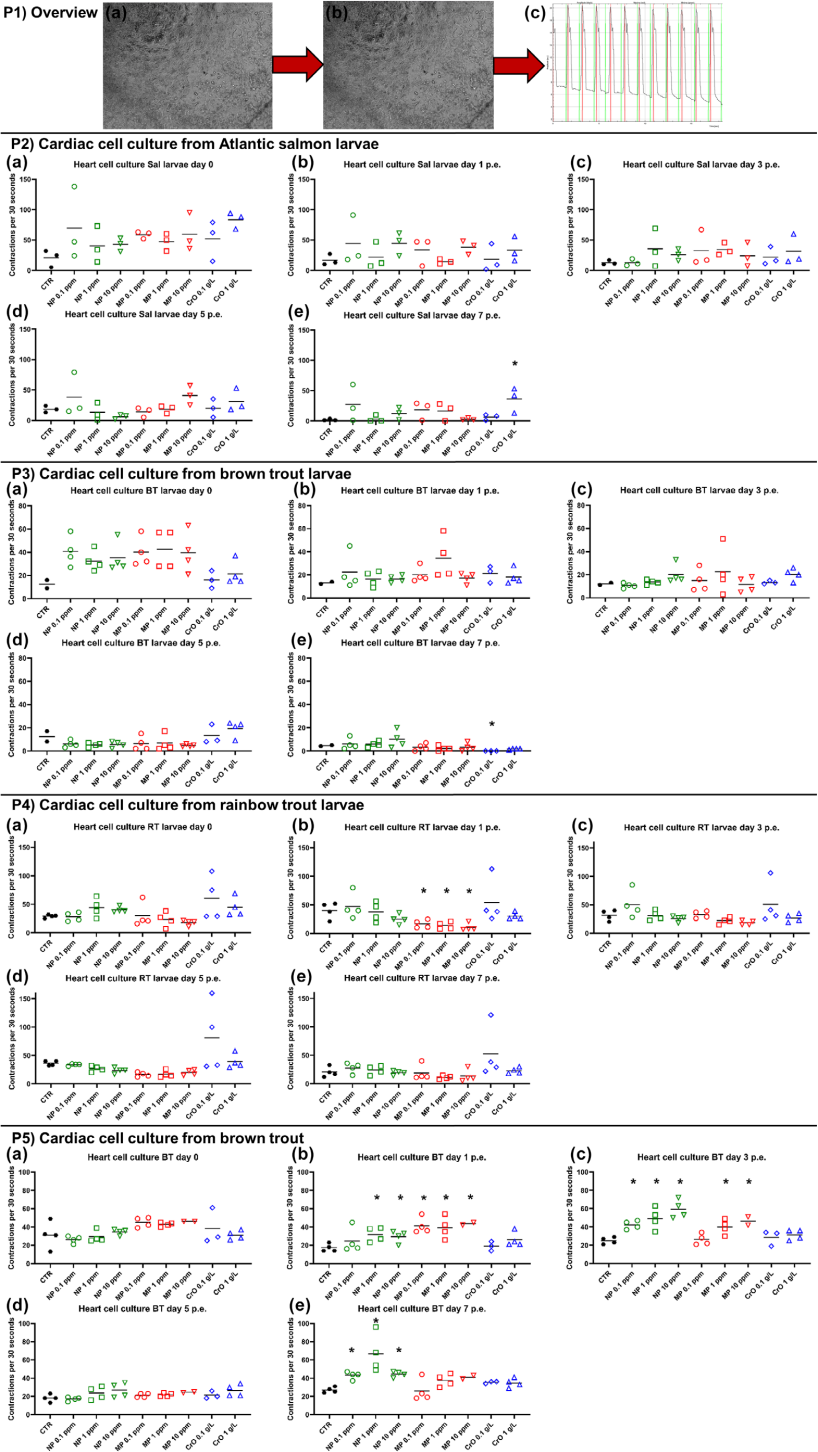
Number of contractions per 30 s in cardiac cell cultures from Atlantic salmon (Panel P2), brown trout (Panel P3) and rainbow trout (Panel P4) larvae and older brown trout (Panel P5) during exposure to microplastic particles (MP), nanoplastic particles (NP) and crude oil (CrO) at different concentrations. Non‐exposed cardiac cell cultures are treated as control (CTR). The overview (Panel P1) illustrates the definition of a contraction of the cardiac cell culture and the method of counting it. A contraction is defined as the transition from a resting, non‐contracting state of the cells (P1a) to contracting state of the cells (P1b), and then back to a resting, non‐contracting state of the cells. Contractions were counted by ImageJ tool *Myocyter* (P1c). Results are presented as scatter plots with all data points and the mean shown. * Indicates statistically significant differences in cardiac contractions of exposed cell cultures compared to unexposed controls within the time point at *p* < .05. Statistical analysis was performed using two‐way ANOVA with multiple comparison test using the Holm‐Sidak method.

The myocardial character of the used cardiac cell cultures was confirmed using immunocytochemistry for alpha‐sarcomeric actin (Figure 2a) and tropomyosin (Figure 2b). At 3 weeks post establishment the majority of the cell cultures were contracting (Figure 2c,d, Video S1). In the results of exposing them to experimental conditions the cultures from salmonid larvae contracted at a rate of 1–160 contractions per 30 s. Exposure of these cultures to plastic particles or crude oil caused a large variation in the rate of contraction. Significant changes in contraction rate were found in all cardiac cell cultures. The greatest differences between control and treatment groups were found in cardiac cell cultures from older brown trout (Figure 3, Panel P5a–e). On day 1 p.e., all cells treated with MNPs showed higher contraction rates compared to control cells (Figure 3, Panel P5b). Nearly the same result was found on day 3 p.e. Only cells treated with 0.1 ppm MP showed no significant difference in contraction rate compared to unexposed cells (Figure 3, Panel P5c). At day 7 p.e., all NP‐treated cells showed significantly higher contraction rates compared to the control (Figure 3, Panel P5e), whereas cells exposed to MP or crude oil contracted at the same rate as untreated controls. In the cardiac cell cultures from larvae of the three salmonid species, significant changes in contraction rate were most frequently found in rainbow trout cultures on day 1 p.e. for all three MP‐treated groups. Here, the contraction rate was significantly lower compared to the control (Figure 3, Panel P4b). In cardiac cell cultures from Atlantic salmon larvae (Figure 3, Panel P2a–e) and brown trout larvae (Figure 3, Panel P3a–e), a significant difference was only observed at day 7 p.e. In Atlantic salmon larvae cultures, cells treated with 1 g/L crude oil contracted at a rate comparable to the control (Figure 3, Panel P2e). In brown trout larval cultures, the contraction rate was significantly lower in cells treated with 0.1 g/L crude oil (Figure 3, Panel P3e). In general, in all cardiac cultures, no cytopathic effect was visible, no accumulation of cells and MNPs occurred, no phagocytosis was observed, and no morphological changes were observed as a result of the treatment. Furthermore, following the preparation of the mixture of crude oil and cell culture medium, no visible droplets of crude oil were observed in the medium. In order to verify the results, in addition to the analyses conducted with the ImageJ tool *Myocyte*r, the contractions were manually counted for the experiments with rainbow trout larvae and brown trout. The results are shown in the Tables S2–S5.

### 3.3 Effects of exposure to MNPs and crude oil on oxidative stress and immune response in Atlantic salmon larval hearts and Atlantic salmon cardiac cell cultures

**FIGURE 4:**
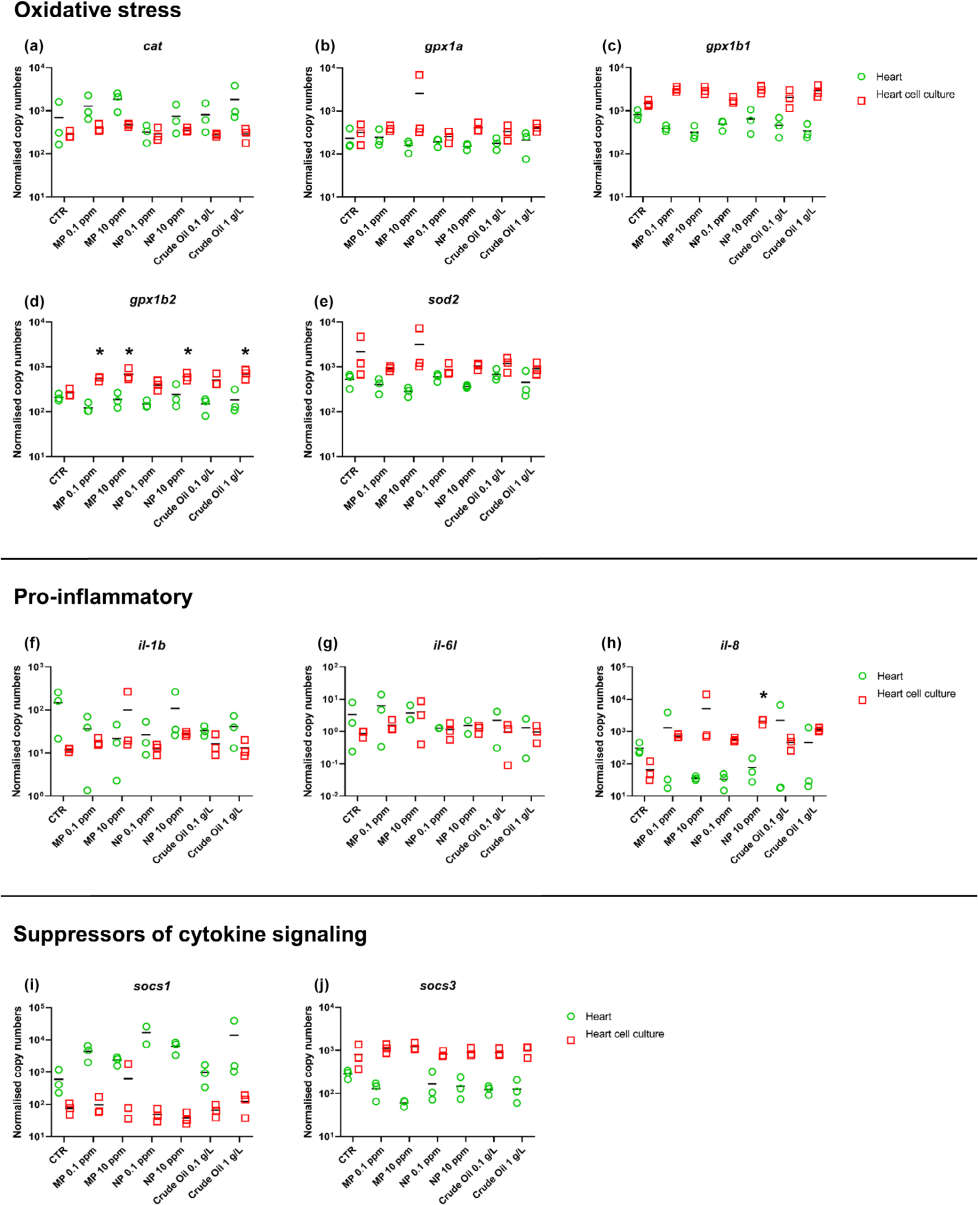
Levels of mRNA encoding Atlantic salmon genes related to oxidative stress (a: *Cat*, b: *Gpx1a*, c: *Gpx1b1*, d: *Gpx1b2*, e: *Sod2*), pro‐inflammatory response (f: *Il‐1b*, g: *Il‐6*, h: *Il‐8*) and suppressors of cytokine signalling (i: *Socs1*, j: *Socs3*) after 7‐day exposure of Atlantic salmon larvae and Atlantic salmon heart cell cultures to different concentrations of micro plastic (MP), nano plastic (NP) particles or crude oil. Results are presented as scatter plots with all data points and the mean shown. Data are presented as normalized copy numbers. * indicates statistically significant differences in expression levels of exposed cardiac cells and exposed hearts of Atlantic salmon larvae compared to unexposed controls at *p* < .05. Statistical analysis was performed using one‐way ANOVA. Where data were not normally distributed, Kruskal‐Wallis one‐way ANOVA on ranks was used.

In general, after 7 days of exposure in Atlantic salmon larval hearts or Atlantic salmon larval cardiac cell cultures there was no strong response to MNPs or crude oil in terms of mRNA expression of genes related to oxidative stress (Figure 4a–e), pro‐inflammatory immune response (Figure 4f–h) and suppressors of cytokine signalling (Figure 4i,j). Only in the cardiac cell culture significant differences were found between the control and treatment groups, focusing on the oxidative stress‐related gene *gpx1b2*. Both treatments with MP and the highest concentration of NP and crude oil resulted in an upregulation of *gpx1b2* (Figure 4d). Only one significant difference was detected in the regulation of pro‐inflammatory genes. The expression of *il‐8* was upregulated in cell cultures exposed to NP at 10 ppm (Figure 4h). No significant differences were observed in the expression of *socs1* (Figure 4i) and *socs3* (Figure 4j) in either hearts or heart cell cultures.

## 4 DISCUSSION

The quantity of studies dealing with the toxicology of environmental stressors is rapidly increasing, and recent findings confirm that the heart is one of the target organs of these stressors. Salmonid species hearts are highly phenotypically plastic organs that can remodel at multiple levels of organization in response to environmental stimuli (Johnston & Gillis, 2018; Keen et al., 2017). The objective of this study was to investigate the impact of environmental stressors, specifically MNPs and crude oil at defined concentrations, on the contraction of the heart and heart cell cultures of salmonid larvae. Additionally, we aimed to determine whether exposure to these stressors induces changes in gene expression of selected genes being markers of oxidative stress, inflammation and cytokine signalling. Our results indicate that after 7 days of exposure, there was no general clear effect of MNPs and crude oil on the heartbeat of salmonid larvae. No significant effect of the contaminants was found on the heart contractions of rainbow trout larvae. In brown trout larvae, a significant difference was found only between the control group and the MP group with a concentration of 1 ppm, where the heartbeat was significantly lower. In Atlantic salmon larvae, a significant increase in heart rate was observed after 3 days post‐exposure under 10 ppm MP treatment, and a significant decrease in heart rate was observed at 5 days post‐exposure. The results indicate that a one‐week exposure to MNPs and crude oil does not cause a measurable change in heart rate for one‐week‐old salmonid larvae. Our results therefore differ from recently published studies in zebrafish embryos. The study showed that after 48 h of exposure to polystyrene NPs with a diameter around 23 nm, the heart rate was reduced at concentrations of 34 ng/L and 34 μg/L. No change was observed at a concentration of 0.04 ng/L (Santos et al., 2024). On the other hand, additional studies on zebrafish embryos at different concentrations ranging from 25 μg/mL to 200 μg/mL or longer exposure times failed to show any significant effect of polystyrene NPs with diameters ranging from 100 to 1000 nm on the heartbeat (Sun et al., 2021; Zhou et al., 2023). Studies investigating the heart rate of *Mallotus villosus* exposed to crude oil failed to show any significant change (Nahrgang et al., 2023). However, previous studies have demonstrated that both MNPs and crude oil can affect fish heart rate, either increasing it (Cheng et al., 2021; Lu, Wu, et al., 2022; Nelson et al., 2017) or decreasing it (Jin et al., 2023; Malafaia et al., 2020; Pitt, Kozal, et al., 2018; Prata et al., 2022). The differences in the results may be attributed to the varying types and doses of environmental stressors used (Lu, Wang, et al., 2022).

Ingestion of toxic substances via feed is a very important route of exposure. The fact that we did not observe any clear dose‐dependent effects of MNP or crude oil exposure on the heart rate of salmonid larvae may be explained by the fact that we used the larvae prior to feeding. Existing studies on the exposure of fish to MPs indicate that there is a complex relationship between the exposure, the uptake of the particles by the fish and their actual effects (Lei et al., 2018). It is assumed that fish primarily ingest plastic particles with their food and that these can overcome the intestinal barrier, enter the bloodstream and accumulate in internal tissues, such as the liver, brain or muscles, of the fish (Ribeiro et al., 2019). However, there is also evidence that the MPs are absorbed via the gills and/or epidermis of fish depending on particle size and length of exposure (Hurt et al., 2020; Park et al., 2020). In contrast to our findings, a study on rainbow trout larvae observed the presence of MPs (diameter 150–180 μm) in 19% of the rainbow trout yolk‐sac larvae following exposure to plastic particles in a 29‐day trial conducted at a concentration of 0.33 mg/L. However, the number of particles present in the digestive tract remained low, ranging from zero to four particles and no mortality was observed during the exposure trial (Jakubowska et al., 2022). In addition, following 14 days of exposure to polystyrene MPs with a size of 0.1 μm, bioaccumulation was already observed after 7 days in various tissues of red tilapia. The bioaccumulation exhibited concentration‐ and time‐dependent effects (Ding et al., 2018). The previous study indicated that 7 days of exposure to polystyrene MPs with a size of 0.1 μm in a low concentration (1 μg/L) is sufficient to enter the body of the fish. In our study, we used even smaller plastic particles and were unable to detect them in the cardiovascular system of the salmonid larvae. This leads to the conclusion that the used plastic particles are still too large to enter the cardiovascular system of the larvae or the used concentrations by us are too low to affect the heartbeat of the larvae. Therefore, we decided to use novel cardiac cell cultures to examine the impact of environmental stressors on the cardiac system at the cellular level. After we exposed cardiac cell cultures of salmonid larvae to MNPs and crude oil at defined concentrations changes in the contraction rate of exposed larval cell cultures were hardly detectable. Only cardiac cell cultures from adult salmonids exhibited a stronger response to the stimulus than those from salmonid larvae. Cultured human cardiomyocytes internalized NPs in a dose‐dependent manner and the exposure to NPs stimulated cardiomyocyte senescence (Wang et al., 2024), but the effect of particle exposure on contraction rate was not assessed. The impact of low concentrations of the ubiquitous contaminant bisphenol A demonstrated that cardiac cells could exhibit a decreased beating rate due to an extension of the contraction and relaxation time (Lamberto et al., 2023). In the present study, when both larval and juvenile salmonid heart cell cultures were exposed to crude oil, no change in contraction and relaxation time was observed.

MNPs can cause inflammatory damage in tissues and organs (Qiao et al., 2019) and trigger oxidative stress (Lu, Wang, et al., 2022; Yang et al., 2020). Cultured human cardiomyocytes internalized nanoparticles, which caused oxidative stress by mitochondrial destabilization and promoted inflammatory responses (Wang et al., 2024). It was of interest whether despite the lack of effects on heartbeat after MNP or crude oil exposure, we could detect any changes in oxidative stress or inflammatory response at the gene expression level in hearts and heart cell cultures from Atlantic salmon larvae. In order to achieve this objective, we selected gene markers that had already been demonstrated to be modulated in response to exposure to environmental stressors in vivo. Despite this we found that exposure to environmental stressors did not significantly alter the expression of suppressors of cytokine signalling and pro‐inflammatory genes. The selected genes that are considered as markers for oxidative stress, also showed little to no significant regulation. The only exception was the oxidative stress related gene *gpx1b2*, which was significantly upregulated in the heart cell cultures exposed to high concentrations of environmental stressors. The data for *gpx1b1* follows a similar pattern, although there are no significant differences. In a study where Atlantic salmon were exposed to dispersed crude oil, it was concluded that crude oil did not induce oxidative stress in the four fish species examined. This may be related to the induction of other antioxidant enzymes, which probably helped to prevent further oxidative damage. Another potential explanation is the selection of biomarkers. It is possible that there are other more sensitive biomarkers for oxidative stress that have not yet been subjected to further research (Sanni et al., 2017). Previous studies have shown that exposure to polystyrene NPs can induce oxidative stress. A study of carp has demonstrated an increase in the activity of CAT, SOD1 and GPX1 following exposure to polystyrene nanoparticles at a concentration of 1000 μg/L for a period of 28 days. Additionally, exposure resulted in significant upregulation of pro‐inflammatory genes (*IL‐1*, *IL‐6*, and *IL‐8*), indicating myocardial inflammation (Wu et al., 2022; Zhang et al., 2023). Other studies have shown that exposure to NPs can reduce the transcript level of *gpx1a*, which can result in stress, inflammation, and tissue destruction (Sun et al., 2016; Wang et al., 2020; Wang et al., 2023). In principle, the activity of CAT and GPX indicates increased ROS production and associated oxidative stress (Chen et al., 2017). In contrast to all of these studies, we could not detect any signs of oxidative stress or inflammation in the heart or in heart cell cultures after 7 days of exposure to environmental stressors in Atlantic salmon larvae. Possible reasons for this are the relatively short exposure time or the fact that salmonid larvae take longer to develop than the commonly used zebrafish larvae. Accordingly, recent articles, which reviewed available literature on field and laboratory studies on the effect of microplastic uptake in freshwater (Parker et al., 2021) and marine fish (Müller, 2021) emphasized the high variability in the response of fish to the challenges. In our study, we used non‐feeding larvae from different salmonid species and cardiomyocyte cultures derived from these larvae. The results indeed showed some species‐specific differences in the response to particle challenges. The observed differences could be attributed to various factors, including the fish's size, age, and trophic level, as well as the characteristics of the particles, such as their size and shape. It appears that fish embryos are more susceptible to the effects of MNPs and crude oil than yolk‐sac larvae, despite the latter being regarded as the most sensitive life stage in fish (Santos et al., 2024). Furthermore, it appears that older fish are more sensitive to environmental stressors than yolk‐sac larvae (Wu et al., 2022; Zhang et al., 2023). Consequently, salmonid larvae seems to be more robust to the challenge and therefore not the optimal developmental state of salmonids to study the effects of environmental stressors (Jakubowska et al., 2020, 2022). Nevertheless, our results demonstrate that short‐term exposure to environmental stressors in salmonid larvae at the selected concentrations is not lethal, does not significantly affect the change in heart rate, and at the cellular level, oxidative stress is very low and there is no evidence of an inflammatory response. The heart cell cultures provide an initial platform for research on the cardiovascular system in vitro, with the potential to contribute to reducing the number of fish used in exposure studies in the long term.

## AUTHOR CONTRIBUTIONS

**Torben Krebs:** Conceptualization; investigation; methodology; data curation; resources; visualization; writing – original draft; writing – review and editing. **Julia Bauer:** Conceptualization; investigation; writing – review and editing; methodology. **Sarah Graff:** Methodology; writing – review and editing; resources. **Lukas Teich:** Investigation; writing – review and editing. **Markus Sterneberg:** Investigation; writing – review and editing. **Marina Gebert:** Conceptualization; resources; project administration; writing – review and editing. **Henrike Seibel:** Writing – review and editing; project administration; resources; conceptualization. **Bettina Seeger:** Methodology; formal analysis; writing – review and editing. **Dieter Steinhagen:** Writing – original draft; supervision; formal analysis; writing – review and editing. **Verena Jung‐Schroers:** Supervision; writing – review and editing. **Mikolaj Adamek:** Conceptualization; funding acquisition; validation; methodology; investigation; writing – original draft; writing – review and editing; visualization; formal analysis; project administration; supervision; data curation; resources.

## FUNDING INFORMATION

This study was funded by the Federal Ministry of Education and Research (BMBF, Germany); Project acronym: SALHEARTCELL; Funding reference number: 161B0865A.

## CONFLICT OF INTEREST STATEMENT

The authors declare that research was conducted in the absence of any commercial or financial relationships that could be construed as a potential conflict of interest.

## Supporting information


Video S1:

Data S1:

## Data Availability

Data available within the article or its supplementary materials.

## References

[jfd14018-bib-0001] Akdogan, Z. , & Guven, B. (2019). Microplastics in the environment: A critical review of current understanding and identification of future research needs. Environmental Pollution, 254, 113011. 10.1016/j.envpol.2019.113011 31404735

[jfd14018-bib-0002] Ali, A. O. , Hohn, C. , Allen, P. J. , Ford, L. , Dail, M. B. , Pruett, S. , & Petrie‐Hanson, L. (2014). The effects of oil exposure on peripheral blood leukocytes and splenic melano‐macrophage centers of Gulf of Mexico fishes. Marine Pollution Bulletin, 79(1–2), 87–93. 10.1016/j.marpolbul.2013.12.036 24405733

[jfd14018-bib-0003] Andrady, A. L. (2022). Weathering and fragmentation of plastic debris in the ocean environment. Marine Pollution Bulletin, 180, 113761. 10.1016/j.marpolbul.2022.113761 35665618

[jfd14018-bib-0004] Aronson, R. B. , Thatje, S. , McClintock, J. B. , & Hughes, K. A. (2011). Anthropogenic impacts on marine ecosystems in Antarctica. Annals of the New York Academy of Sciences, 1223, 82–107. 10.1111/j.1749-6632.2010.05926.x 21449967

[jfd14018-bib-0005] Barnes, D. K. A. , Galgani, F. , Thompson, R. C. , & Barlaz, M. (2009). Accumulation and fragmentation of plastic debris in global environments. Philosophical Transactions of the Royal Society, B: Biological Sciences, 364(1526), 1985–1998. 10.1098/rstb.2008.0205 PMC2873009 19528051

[jfd14018-bib-0006] Besseling, E. , Redondo‐Hasselerharm, P. , Foekema, E. M. , & Koelmans, A. A. (2019). Quantifying ecological risks of aquatic micro‐ and nanoplastic. Critical Reviews in Environmental Science and Technology, 49(1), 32–80. 10.1080/10643389.2018.1531688

[jfd14018-bib-0007] Borrelle, S. B. , Ringma, J. , Law, K. L. , Monnahan, C. C. , Lebreton, L. , McGivern, A. , Murphy, E. , Jambeck, J. , Leonard, G. H. , Hilleary, M. A. , Eriksen, M. , Possingham, H. P. , De Frond, H. , Gerber, L. R. , Polidoro, B. , Tahir, A. , Bernard, M. , Mallos, N. , Barnes, M. , & Rochman, C. M. (2020). Predicted growth in plastic waste exceeds efforts to mitigate plastic pollution. Science, 369(6510), 1515–1518. 10.1126/science.aba3656 32943526

[jfd14018-bib-0008] Browne, M. A. , Galloway, T. , & Thompson, R. (2007). Microplastic—An emerging contaminant of potential concern? Integrated Environmental Assessment and Management, 3(4), 559–561. 10.1002/ieam.5630030412 18046805

[jfd14018-bib-0009] Chen, J. , Liang, Q. , Zheng, Y. , Lei, Y. , Gan, X. , Mei, H. , Bai, C. , Wang, H. , Ju, J. , Dong, Q. , & Song, Y. (2024). Polystyrene nanoplastics induced size‐dependent developmental and neurobehavioral toxicities in embryonic and juvenile zebrafish. Aquatic Toxicology, 267, 106842. 10.1016/j.aquatox.2024.106842 38266469

[jfd14018-bib-0010] Chen, J. , Zhang, W. , Wan, Z. , Li, S. , Huang, T. , & Fei, Y. (2019). Oil spills from global tankers: Status review and future governance. Journal of Cleaner Production, 227, 20–32. 10.1016/j.jclepro.2019.04.020

[jfd14018-bib-0011] Chen, Q. , Gundlach, M. , Yang, S. , Jiang, J. , Velki, M. , Yin, D. , & Hollert, H. (2017). Quantitative investigation of the mechanisms of microplastics and nanoplastics toward zebrafish larvae locomotor activity. Science of the Total Environment, 584‐585, 1022–1031. 10.1016/j.scitotenv.2017.01.156 28185727

[jfd14018-bib-0012] Cheng, H. , Feng, Y. , Duan, Z. , Duan, X. , Zhao, S. , Wang, Y. , Gong, Z. , & Wang, L. (2021). Toxicities of microplastic fibers and granules on the development of zebrafish embryos and their combined effects with cadmium. Chemosphere, 269, 128677. 10.1016/j.chemosphere.2020.128677 33657748

[jfd14018-bib-0013] Couillard, C. M. , Lee, K. , Légaré, B. , & King, T. L. (2005). Effect of dispersant on the composition of the water‐accommodated fraction of crude oil and its toxicity to larval marine fish. Environmental Toxicology and Chemistry, 24(6), 1496–1504. 10.1897/04-267r.1 16117127

[jfd14018-bib-0014] Ding, J. , Zhang, S. , Razanajatovo, R. M. , Zou, H. , & Zhu, W. (2018). Accumulation, tissue distribution, and biochemical effects of polystyrene microplastics in the freshwater fish red tilapia (*Oreochromis niloticus*). Environmental Pollution, 238, 1–9. 10.1016/j.envpol.2018.03.001 29529477

[jfd14018-bib-0015] Fodrie, F. J. , Able, K. W. , Galvez, F. , Heck, K. L., Jr. , Jensen, O. P. , López‐Duarte, P. C. , Martin, C. W. , Turner, R. E. , & Whitehead, A. (2014). Integrating organismal and population responses of estuarine fishes in Macondo spill research. Bioscience, 64(9), 778–788. 10.1093/biosci/biu123

[jfd14018-bib-0016] Fu, X. , Han, H. , Yang, H. , Xu, B. , Dai, W. , Liu, L. , He, T. , Du, X. , & Pei, X. (2024). Nrf2‐mediated ferroptosis of spermatogenic cells involved in male reproductive toxicity induced by polystyrene nanoplastics in mice. Journal of Zhejiang University. Science. B, 25(4), 307–323. 10.1631/jzus.B2300138 38584093 PMC11009441

[jfd14018-bib-0017] Gigault, J. , Halle, A. T. , Baudrimont, M. , Pascal, P.‐Y. , Gauffre, F. , Phi, T.‐L. , El Hadri, H. , Grassl, B. , & Reynaud, S. (2018). Current opinion: What is a nanoplastic? Environmental Pollution, 235, 1030–1034. 10.1016/j.envpol.2018.01.024 29370948

[jfd14018-bib-0018] Hjermann, D. Ø. , Melsom, A. , Dingsør, G. E. , Durant, J. M. , Eikeset, A. M. , Røed, L. P. , Ottersen, G. , Storvik, G. , & Stenseth, N. C. (2007). Fish and oil in the Lofoten–Barents Sea system: Synoptic review of the effect of oil spills on fish populations. Marine Ecology Progress Series, 339, 283–299.

[jfd14018-bib-0019] Huang, W. , Yang, Y. , Tang, S. , Yin, H. , Yu, X. , Yu, Y. , & Wei, K. (2024). The combined toxicity of polystyrene nano/micro‐plastics and triphenyl phosphate (TPHP) on HepG2 cells. Ecotoxicology and Environmental Safety, 279, 116489. 10.1016/j.ecoenv.2024.116489 38776781

[jfd14018-bib-0020] Hurt, R. , O'Reilly, C. M. , & Perry, W. L. (2020). Microplastic prevalence in two fish species in two U.S. reservoirs. Limnology and Oceanography Letters, 5(1), 147–153. 10.1002/lol2.10140

[jfd14018-bib-0021] Incardona, J. P. , & Scholz, N. L. (2016). The influence of heart developmental anatomy on cardiotoxicity‐based adverse outcome pathways in fish. Aquatic Toxicology, 177, 515–525. 27447099 10.1016/j.aquatox.2016.06.016

[jfd14018-bib-0022] Jakubowska, M. , Białowąs, M. , Stankevičiūtė, M. , Chomiczewska, A. , Jonko‐Sobuś, K. , Pažusienė, J. , Hallmann, A. , Bučaitė, A. , & Urban‐Malinga, B. (2022). Effects of different types of primary microplastics on early life stages of rainbow trout (*Oncorhynchus mykiss*). Science of the Total Environment, 808, 151909. 10.1016/j.scitotenv.2021.151909 34838922

[jfd14018-bib-0023] Jakubowska, M. , Białowąs, M. , Stankevičiūtė, M. , Chomiczewska, A. , Pažusienė, J. , Jonko‐Sobuś, K. , Hallmann, A. , & Urban‐Malinga, B. (2020). Effects of chronic exposure to microplastics of different polymer types on early life stages of sea trout Salmo trutta. Science of the Total Environment, 740, 139922. 10.1016/j.scitotenv.2020.139922 32927534

[jfd14018-bib-0024] Jin, F. , Wang, Y. , Yu, F. , Liu, X. , Zhang, M. , Li, Z. , Yao, Z. , Cong, Y. , & Wang, J. (2023). Acute and chronic effects of crude oil water‐accommodated fractions on the early life stages of marine medaka (Oryzias melastigma, McClelland, 1839). Toxics, 11(3), 236. 10.3390/toxics11030236 36977001 PMC10053065

[jfd14018-bib-0025] Johnston, E. F. , & Gillis, T. E. (2018). Transforming growth factor‐β1 induces differentiation of rainbow trout (*Oncorhynchus mykiss*) cardiac fibroblasts into myofibroblasts. Journal of Experimental Biology, 221(24), jeb189167. 10.1242/jeb.189167 30397172

[jfd14018-bib-0026] Keen, A. N. , Klaiman, J. M. , Shiels, H. A. , & Gillis, T. E. (2017). Temperature‐induced cardiac remodelling in fish. Journal of Experimental Biology, 220(2), 147–160. 10.1242/jeb.128496 27852752 PMC5278617

[jfd14018-bib-0027] Kim, Y.‐N. , Yoon, J.‐H. , & Kim, K.‐H. J. (2020). Microplastic contamination in soil environment – A review. Soil Science Annual, 71(4), 300–308. 10.37501/soilsa/131646

[jfd14018-bib-0028] Lamberto, F. , Shashikadze, B. , Elkhateib, R. , Lombardo, S. D. , Horánszky, A. , Balogh, A. , Kistamás, K. , Zana, M. , Menche, J. , Fröhlich, T. , & Dinnyés, A. (2023). Low‐dose bisphenol a exposure alters the functionality and cellular environment in a human cardiomyocyte model. Environmental Pollution, 335, 122359. 10.1016/j.envpol.2023.122359 37567409

[jfd14018-bib-0029] Lei, L. , Wu, S. , Lu, S. , Liu, M. , Song, Y. , Fu, Z. , Shi, H. , Raley‐Susman, K. M. , & He, D. (2018). Microplastic particles cause intestinal damage and other adverse effects in zebrafish Danio rerio and nematode Caenorhabditis elegans. Sci Total Environ, 619‐620, 1–8. 10.1016/j.scitotenv.2017.11.103 29136530

[jfd14018-bib-0030] Li, J. , Weng, H. , Liu, S. , Li, F. , Xu, K. , Wen, S. , Chen, X. , Li, C. , Nie, Y. , Liao, B. , Wu, J. , Kantawong, F. , Xie, X. , Yu, F. , & Li, G. (2024). Embryonic exposure of polystyrene nanoplastics affects cardiac development. Science of the Total Environment, 906, 167406. 10.1016/j.scitotenv.2023.167406 37769743

[jfd14018-bib-0031] Lu, J. , Wang, W. , Xu, W. , Zhang, C. , Zhang, C. , Tao, L. , Li, Z. , & Zhang, Y. (2022). Induction of developmental toxicity and cardiotoxicity in zebrafish embryos by Emamectin benzoate through oxidative stress. Science of the Total Environment, 825, 154040. 35196543 10.1016/j.scitotenv.2022.154040

[jfd14018-bib-0032] Lu, J. , Wu, J. , Gong, L. , Cheng, Y. , Yuan, Q. , & He, Y. (2022). Combined toxicity of polystyrene microplastics and sulfamethoxazole on zebrafish embryos. Environmental Science and Pollution Research, 29, 1–10. 34714475 10.1007/s11356-021-17198-8

[jfd14018-bib-0033] Malafaia, G. , de Souza, A. M. , Pereira, A. C. , Gonçalves, S. , da Costa Araújo, A. P. , Ribeiro, R. X. , & Rocha, T. L. (2020). Developmental toxicity in zebrafish exposed to polyethylene microplastics under static and semi‐static aquatic systems. Science of the Total Environment, 700, 134867. 10.1016/j.scitotenv.2019.134867 31706091

[jfd14018-bib-0034] Martínez‐Gómez, C. , Campillo, J. A. , Benedicto, J. , Fernández, B. , Valdés, J. , García, I. , & Sánchez, F. (2006). Monitoring biomarkers in fish (Lepidorhombus boscii and Callionymus lyra) from the northern Iberian shelf after the prestige oil spill. Marine Pollution Bulletin, 53(5), 305–314. 10.1016/j.marpolbul.2006.03.010 16698047

[jfd14018-bib-0035] Meador, J. P. , & Nahrgang, J. (2019). Characterizing crude oil toxicity to early‐life stage fish based on a complex mixture: Are we making unsupported assumptions? Environmental Science & Technology, 53(19), 11080–11092. 10.1021/acs.est.9b02889 31503459

[jfd14018-bib-0036] Mendoza, L. M. R. , & Balcer, M. (2019). Microplastics in freshwater environments: A review of quantification assessment. TrAC Trends in Analytical Chemistry, 113, 402–408.

[jfd14018-bib-0037] Müller, C. (2021). Not as bad as it seems? A literature review on the case of microplastic uptake in fish. Frontiers in Marine Science, 8, 672768. 10.3389/fmars.2021.672768

[jfd14018-bib-0038] Murawski, S. A. , Hollander, D. J. , Gilbert, S. , & Gracia, A. (2020). Deepwater oil and gas production in the Gulf of Mexico and related global trends. In S. A. Murawski , C. H. Ainsworth , S. Gilbert , D. J. Hollander , C. B. Paris , M. Schlüter , & D. L. Wetzel (Eds.), Scenarios and responses to future deep oil spills: Fighting the next war (pp. 16–32). Springer International Publishing.

[jfd14018-bib-0039] Nahrgang, J. , Granlund, C. , Bender, M. L. , Sørensen, L. , Greenacre, M. , & Frantzen, M. (2023). No observed developmental effects in early life stages of capelin (Mallotus villosus) exposed to a water‐soluble fraction of crude oil during embryonic development. Journal of Toxicology and Environmental Health. Part A, 86(12), 404–419. 10.1080/15287394.2023.2209115 37171367

[jfd14018-bib-0040] Nelson, D. , Stieglitz, J. D. , Cox, G. K. , Heuer, R. M. , Benetti, D. D. , Grosell, M. , & Crossley, D. A. (2017). Cardio‐respiratory function during exercise in the cobia, Rachycentron canadum: The impact of crude oil exposure. Comparative Biochemistry and Physiology Part C: Toxicology & Pharmacology, 201, 58–65. 10.1016/j.cbpc.2017.08.006 28923244

[jfd14018-bib-0041] Park, T.‐J. , Lee, S.‐H. , Lee, M.‐S. , Lee, J.‐K. , Lee, S.‐H. , & Zoh, K.‐D. (2020). Occurrence of microplastics in the Han River and riverine fish in South Korea. Science of the Total Environment, 708, 134535. 10.1016/j.scitotenv.2019.134535 31806294

[jfd14018-bib-0042] Parker, B. , Andreou, D. , Green, I. D. , & Britton, J. R. (2021). Microplastics in freshwater fishes: Occurrence, impacts and future perspectives. Fish and Fisheries, 22(3), 467–488. 10.1111/faf.12528

[jfd14018-bib-0043] Pitt, J. A. , Kozal, J. S. , Jayasundara, N. , Massarsky, A. , Trevisan, R. , Geitner, N. , Wiesner, M. , Levin, E. D. , & Di Giulio, R. T. (2018). Uptake, tissue distribution, and toxicity of polystyrene nanoparticles in developing zebrafish (Danio rerio). Aquatic Toxicology, 194, 185–194. 10.1016/j.aquatox.2017.11.017 29197232 PMC6959514

[jfd14018-bib-0044] Pitt, J. A. , Trevisan, R. , Massarsky, A. , Kozal, J. S. , Levin, E. D. , & Di Giulio, R. T. (2018). Maternal transfer of nanoplastics to offspring in zebrafish (Danio rerio): A case study with nanopolystyrene. Science of the Total Environment, 643, 324–334. 10.1016/j.scitotenv.2018.06.186 29940444 PMC7012458

[jfd14018-bib-0045] PlasticsEurope . (2023). Plastics—The the fast Facts 2023. https://plasticseurope.org/knowledge‐hub/plastics‐the‐fast‐facts‐2023/

[jfd14018-bib-0046] Prata, J. C. , Venâncio, C. , Girão, A. V. , da Costa, J. P. , Lopes, I. , Duarte, A. C. , & Rocha‐Santos, T. (2022). Effects of virgin and weathered polystyrene and polypropylene microplastics on Raphidocelis subcapitata and embryos of Danio rerio under environmental concentrations. Science of the Total Environment, 816, 151642. 10.1016/j.scitotenv.2021.151642 34822904

[jfd14018-bib-0047] Qiao, R. , Sheng, C. , Lu, Y. , Zhang, Y. , Ren, H. , & Lemos, B. (2019). Microplastics induce intestinal inflammation, oxidative stress, and disorders of metabolome and microbiome in zebrafish. Science of the Total Environment, 662, 246–253. 30690359 10.1016/j.scitotenv.2019.01.245

[jfd14018-bib-0048] Ramachandran, S. D. , Hodson, P. V. , Khan, C. W. , & Lee, K. (2004). Oil dispersant increases PAH uptake by fish exposed to crude oil. Ecotoxicology and Environmental Safety, 59(3), 300–308. 10.1016/j.ecoenv.2003.08.018 15388269

[jfd14018-bib-0049] Ribeiro, F. , O'Brien, J. W. , Galloway, T. , & Thomas, K. V. (2019). Accumulation and fate of nano‐ and micro‐plastics and associated contaminants in organisms. TrAC Trends in Analytical Chemistry, 111, 139–147. 10.1016/j.trac.2018.12.010

[jfd14018-bib-0050] Sander, V. , Suñe, G. , Jopling, C. , Morera, C. , & Izpisua Belmonte, J. C. (2013). Isolation and in vitro culture of primary cardiomyocytes from adult zebrafish hearts. Nature Protocols, 8(4), 800–809. 10.1038/nprot.2013.041 23538883

[jfd14018-bib-0051] Sanni, S. , Björkblom, C. , Jonsson, H. , Godal, B. F. , Liewenborg, B. , Lyng, E. , & Pampanin, D. M. (2017). I: Biomarker quantification in fish exposed to crude oil as input to species sensitivity distributions and threshold values for environmental monitoring. Marine Environmental Research, 125, 10–24. 10.1016/j.marenvres.2016.12.002 28038348

[jfd14018-bib-0052] Santos, A. L. , Rodrigues, L. C. , Rodrigues, C. C. , Cirqueira, F. , Malafaia, G. , & Rocha, T. L. (2024). Polystyrene nanoplastics induce developmental impairments and vasotoxicity in zebrafish (Danio rerio). Journal of Hazardous Materials, 464, 132880. 10.1016/j.jhazmat.2023.132880 37956561

[jfd14018-bib-0053] Schein, A. , Scott, J. A. , Mos, L. , & Hodson, P. V. (2009). Oil dispersion increases the apparent bioavailability and toxicity of diesel to rainbow trout (*Oncorhynchus mykiss*). Environmental Toxicology and Chemistry, 28(3), 595–602. 10.1897/08-315.1 18939894

[jfd14018-bib-0054] Sørhus, E. , Donald, C. E. , da Silva, D. , Thorsen, A. , Karlsen, Ø. , & Meier, S. (2021). Untangling mechanisms of crude oil toxicity: Linking gene expression, morphology and PAHs at two developmental stages in a cold‐water fish. Science of the Total Environment, 757, 143896. 10.1016/j.scitotenv.2020.143896 33316527

[jfd14018-bib-0055] Sun, M. , Ding, R. , Ma, Y. , Sun, Q. , Ren, X. , Sun, Z. , & Duan, J. (2021). Cardiovascular toxicity assessment of polyethylene nanoplastics on developing zebrafish embryos. Chemosphere, 282, 131124. 10.1016/j.chemosphere.2021.131124 34374342

[jfd14018-bib-0056] Sun, Y. , Zhang, G. , He, Z. , Wang, Y. , Cui, J. , & Li, Y. (2016). Effects of copper oxide nanoparticles on developing zebrafish embryos and larvae. International Journal of Nanomedicine, 11, 905–918. 27022258 10.2147/IJN.S100350 PMC4788362

[jfd14018-bib-0057] Sun, Y. , Zhao, X. , Sui, Q. , Sun, X. , Zhu, L. , Booth, A. M. , Chen, B. , Qu, K. , & Xia, B. (2024). Polystyrene nanoplastics affected the nutritional quality of Chlamys farreri through disturbing the function of gills and physiological metabolism: Comparison with microplastics. Science of the Total Environment, 910, 168457. 10.1016/j.scitotenv.2023.168457 37981153

[jfd14018-bib-0058] Thompson, R. (2017). Future of the sea: Plastic pollution. Foresight, Government Office for Science.

[jfd14018-bib-0059] Torres‐Ruiz, M. , De la Vieja, A. , de Alba Gonzalez, M. , Esteban Lopez, M. , Castaño Calvo, A. , & Cañas Portilla, A. I. (2021). Toxicity of nanoplastics for zebrafish embryos, what we know and where to go next. Science of the Total Environment, 797, 149125. 10.1016/j.scitotenv.2021.149125 34346375

[jfd14018-bib-0060] Veneman, W. J. , Spaink, H. P. , Brun, N. R. , Bosker, T. , & Vijver, M. G. (2017). Pathway analysis of systemic transcriptome responses to injected polystyrene particles in zebrafish larvae. Aquatic Toxicology, 190, 112–120. 10.1016/j.aquatox.2017.06.014 28704660

[jfd14018-bib-0061] Wang, K. , Du, Y. , Li, P. , Guan, C. , Zhou, M. , Wu, L. , Liu, Z. , & Huang, Z. (2024). Nanoplastics causes heart aging/myocardial cell senescence through the Ca^2+^/mtDNA/cGAS‐STING signaling cascade. Journal of Nanobiotechnology, 22(1), 96. 10.1186/s12951-024-02375-x 38448951 PMC10918962

[jfd14018-bib-0062] Wang, L. , Wang, Q. , Xiao, G. , Chen, G. , Han, L. , & Hu, T. (2020). Adverse effect of cylindrospermopsin on embryonic development in zebrafish (Danio rerio). Chemosphere, 241, 125060. 31629243 10.1016/j.chemosphere.2019.125060

[jfd14018-bib-0063] Wang, Q. , Chen, G. , Tian, L. , Kong, C. , Gao, D. , Chen, Y. , Junaid, M. , & Wang, J. (2023). Neuro‐ and hepato‐toxicity of polystyrene nanoplastics and polybrominated diphenyl ethers on early life stages of zebrafish. Science of the Total Environment, 857, 159567. 10.1016/j.scitotenv.2022.159567 36272476

[jfd14018-bib-0064] Wu, H. , Guo, J. , Yao, Y. , & Xu, S. (2022). Polystyrene nanoplastics induced cardiomyocyte apoptosis and myocardial inflammation in carp by promoting ROS production. Fish & Shellfish Immunology, 125, 1–8. 10.1016/j.fsi.2022.04.048 35504440

[jfd14018-bib-0065] Wu, Y. , Li, L. , Tang, L. , Peijnenburg, W. , Zhang, H. , Xie, D. , Geng, R. , Zheng, T. , Bi, L. , Wei, X. , Chae, H. J. , Wang, L. , Zhao, L. , Li, B. , & Zheng, Q. (2024). Ototoxicity of polystyrene nanoplastics in mice, HEI‐OC1 cells and zebrafish. Frontiers in Molecular Neuroscience, 17, 1345536. 10.3389/fnmol.2024.1345536 38440220 PMC10909942

[jfd14018-bib-0066] Yang, H. , Xiong, H. , Mi, K. , Xue, W. , Wei, W. , & Zhang, Y. (2020). Toxicity comparison of nano‐sized and micron‐sized microplastics to goldfish Carassius auratus larvae. Journal of Hazardous Materials, 388, 122058. 31951993 10.1016/j.jhazmat.2020.122058

[jfd14018-bib-0067] Yang, W. , Gao, P. , Li, H. , Huang, J. , Zhang, Y. , Ding, H. , & Zhang, W. (2021). Mechanism of the inhibition and detoxification effects of the interaction between nanoplastics and microalgae Chlorella pyrenoidosa. Science of the Total Environment, 783, 146919. 10.1016/j.scitotenv.2021.146919 33866172

[jfd14018-bib-0068] Zhang, Q. , Wang, F. , Xu, S. , Cui, J. , Li, K. , Shiwen, X. , & Guo, M.‐Y. (2023). Polystyrene microplastics induce myocardial inflammation and cell death via the TLR4/NF‐κB pathway in carp. Fish & Shellfish Immunology, 135, 108690. 10.1016/j.fsi.2023.108690 36944415

[jfd14018-bib-0069] Zhou, R. , Zhou, D. , Yang, S. , Shi, Z. , Pan, H. , Jin, Q. , & Ding, Z. (2023). Neurotoxicity of polystyrene nanoplastics with different particle sizes at environment‐related concentrations on early zebrafish embryos. Science of the Total Environment, 872, 162096. 10.1016/j.scitotenv.2023.162096 36791853

