# Supplementary material for: Use of cardiac cell cultures from salmonids to measure the cardiotoxic effect of environmental pollutants
Source: J Fish Dis. 2024 Sep 29;48(1):e14018. doi: 10.1111/jfd.14018 (PMC11646967; doi:10.1111/jfd.14018)
Supplement: Supplementary file 2 — Data S1: [file JFD-48-e14018-s002.docx]

**Supplementary data:**

**Supplementary Table 1:** Primer sequences used in this work. All primer sequences were used in RT-qPCR gene mRNA expression analysis.

| **Gene** | **Forward Primer** | **Reverse Primer** |
| --- | --- | --- |
| cat | TGATGTCACACAGGTGCGTA | GTGGGCTCAGTGTTGTTGAG |
| coilp84 | GCTCATTTGAGGAGAAGGAGGATG | CTGGCGATGCTGTTCCTGAG |
| ef1α | TGGGCTGGTTCAAGGGATGG | CTGGAGGGGCAGACGAAGG |
| gpx1a | AATGTGGCGTCACTCTGAGG | CAATTCTCCTGATGGCCAAA |
| gpx1b1 | CGAGCTCCATGAACGGTACG | TGCTTCCCGTTCACATCCAC |
| gpx1b2 | TCGGACATCAGGAGAACTGC | TCCTTCCCATTCACATCCAC |
| il-1b | GTATCCCATCACCCCATCAC | TTCTTCCACAGCACTCTCCA |
| il-6l | GGCTGAATACCCACAATCCA | TAGACACCTCACCCAGCACA |
| il-8 | CACTGAGATCATTGCCACTCTGA | ATGACCCTCTTGACCCACGG |
| socs1 | GATTAATACCGCTGGGATTCTGTG | CTCTCCCATCGCTACACAGTTCC |
| socs3 | GAACAACACAAGATATCAAGCTCAAGG | GAAGGTCTTGTAACGGTGAGGCAG |
| sod2 | TCCCTGACCTGACCTACGAC | GGCCTCCTCCATTAAACCTC |
| 39SL40 | CCCAGTATGAGGCACCTGAAGG | GTTAATGCTGCCACCCTCTCAC |


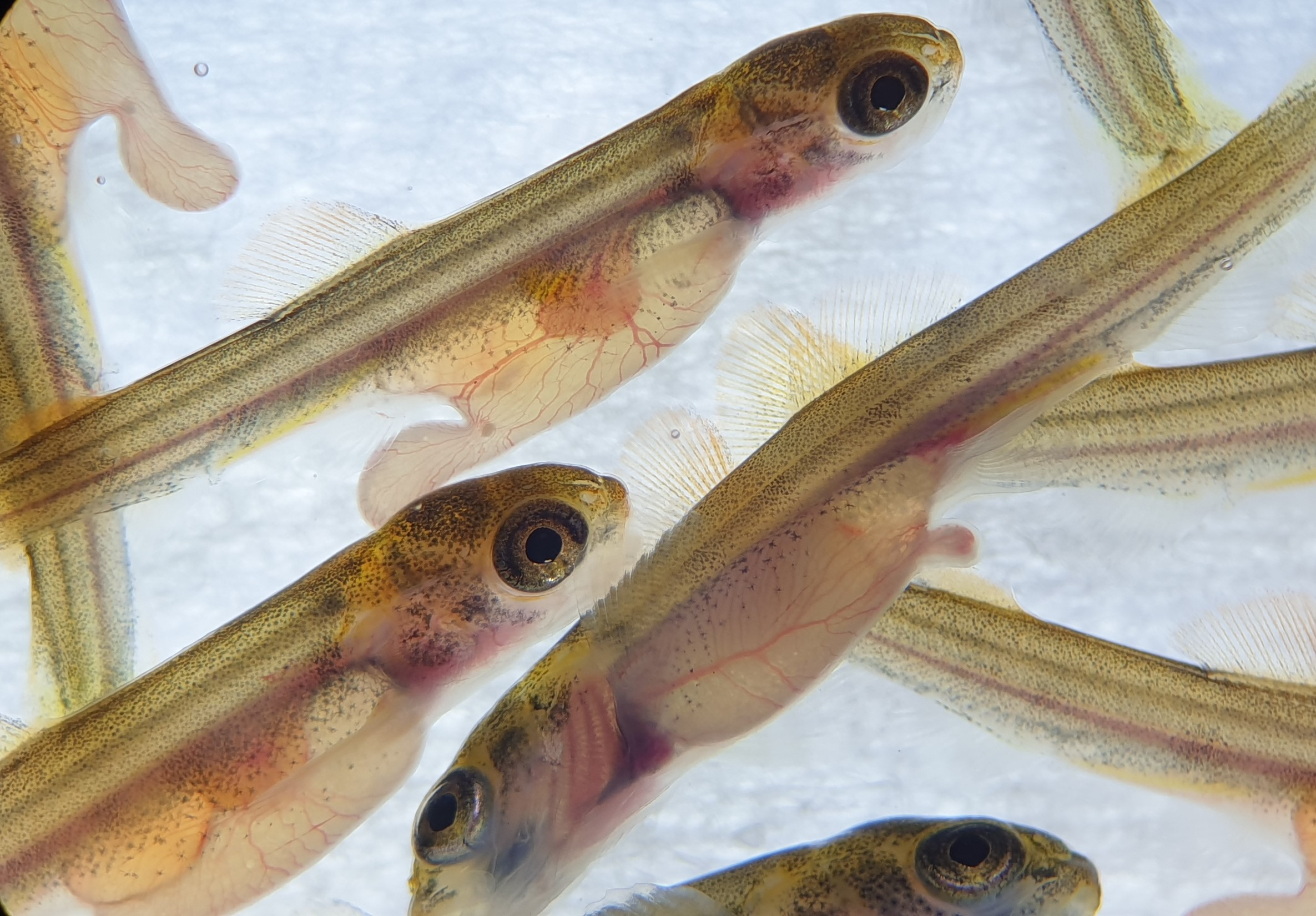


**Supplementary Figure 1:** Example of Atlantic salmon larvae used for primary cell culture and *in vivo* exposure experiment


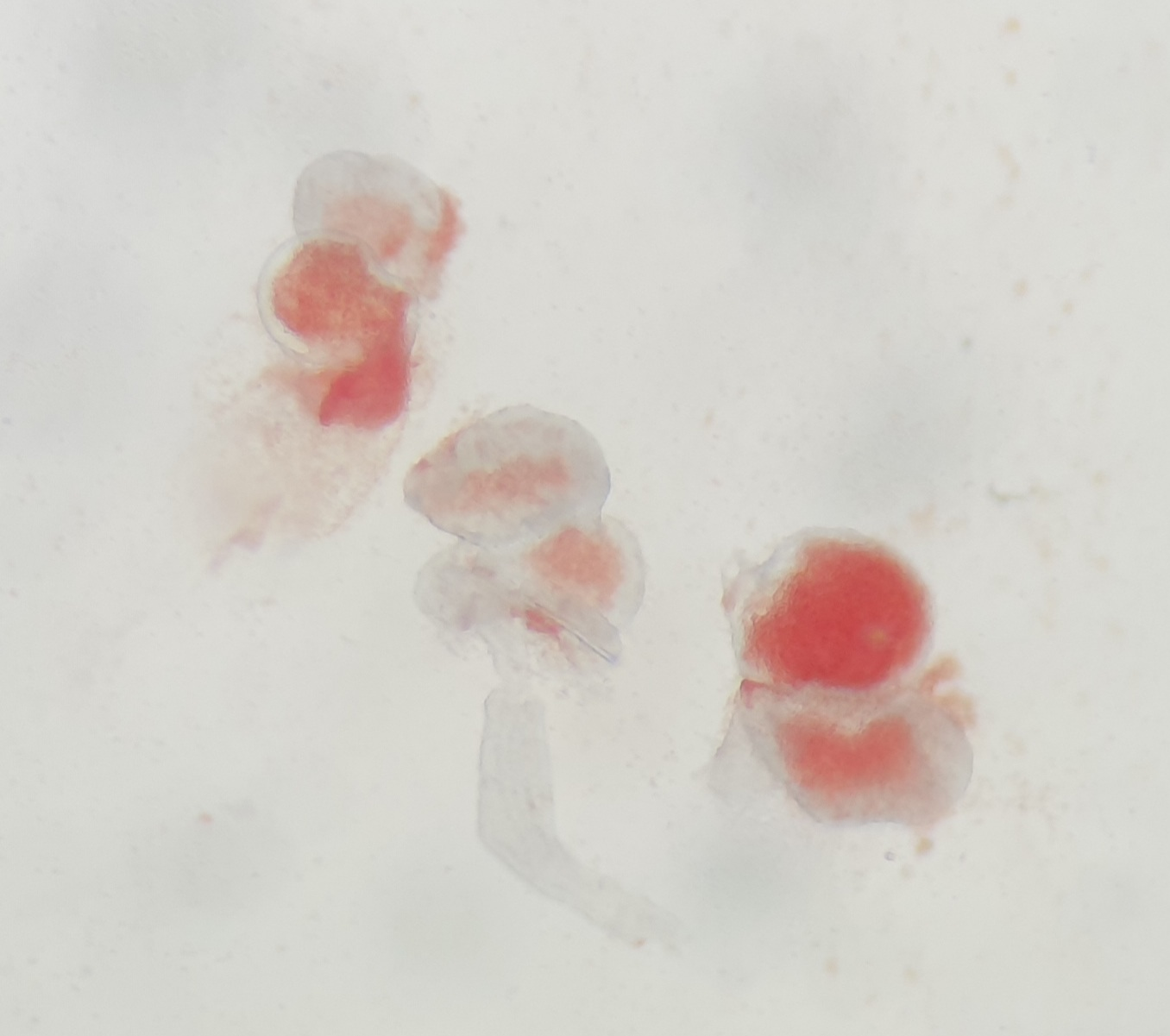


**Supplementary Figure 2:** Example of rainbow trout larval hearts extracted for primary cell culture


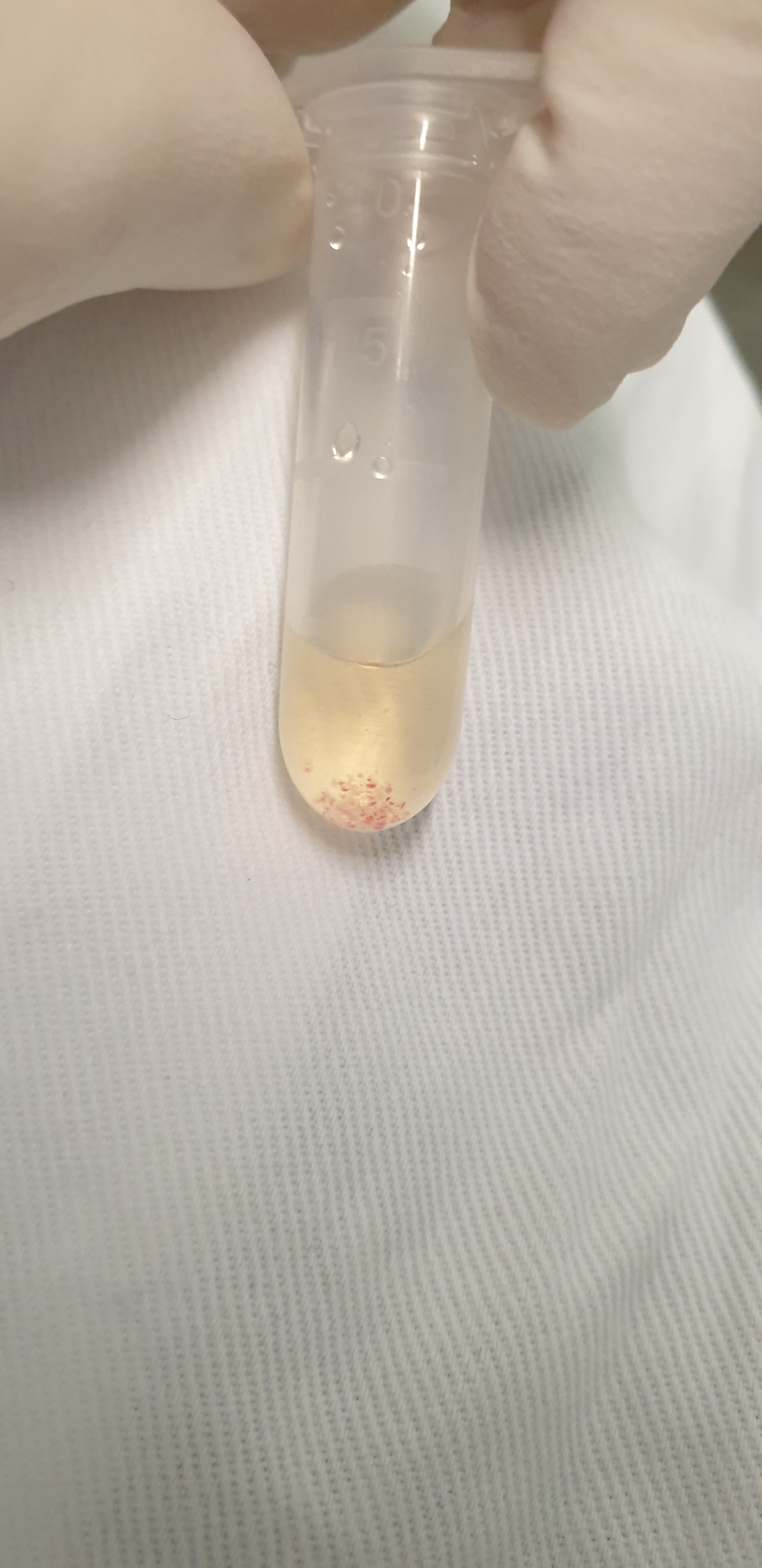


**Supplementary Figure 3:** Example of larval hearts transferred to 2 mL Eppendorf tube for washing with perfusion buffer


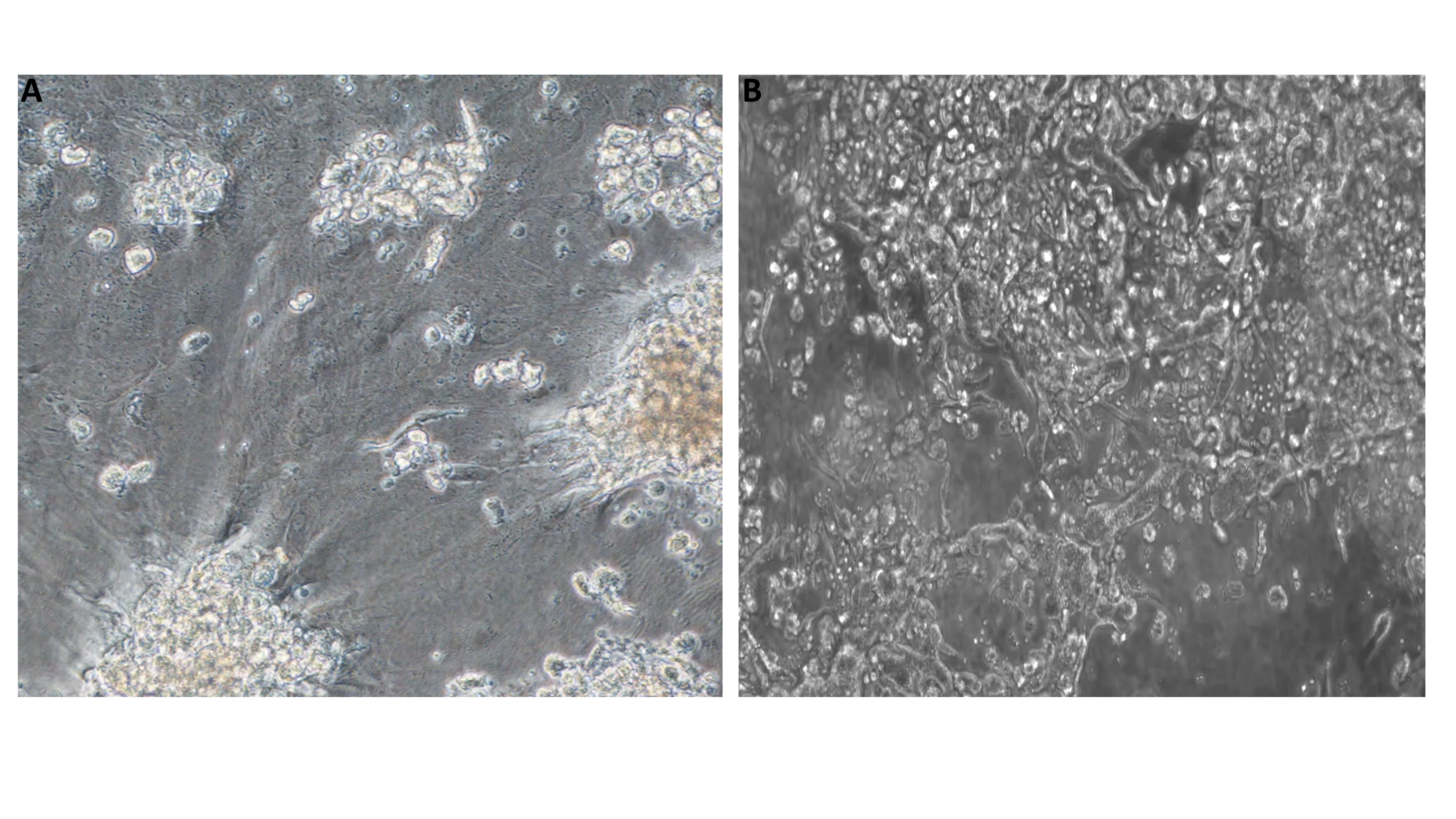


**Supplementary Figure 4:** Examples of three-week-old primary rainbow trout (A) and brown trout (B) cardiac cell cultures.

**Supplementary Table 2: Results of a hand-counted analysis of the contractions of rainbow trout larvae cardiac cell cultures**

**Supplementary Table 3: Results of analysis of the contractions of rainbow trout larvae cardiac cell cultures counted by ImageJ tool *Myocyter***

**Supplementary Table 4: Results of a hand-counted analysis of the contractions of brown trout cardiac cell cultures**

**Supplementary Table 5: Results of analysis of the contractions of brown trout cardiac cell cultures counted by ImageJ tool *Myocyter***
